# Inflammation as a harbinger of death: a retrospective cohort study of preoperative biomarker risk stratification in orthopedic trauma

**DOI:** 10.55730/1300-0144.6141

**Published:** 2026-02-04

**Authors:** Serkan AYDIN, Burhan KURTULUŞ

**Affiliations:** 1 Department of Orthopedics and Traumatology, Ministry of Health Etlik City Hospital, Ankara, Turkiye; 2 Department of Orthopedics and Traumatology, Ankara Dışkapı Yıldırım Beyazıt Education and Research Hospital, Ankara, Turkiye

**Keywords:** Femur fracture, mortality, inflammatory markers, prognosis, surgery, risk stratification

## Abstract

**Background/aim:**

This study aimed to evaluate the prognostic significance of preoperative inflammatory markers including the neutrophil-to-lymphocyte ratio (NLR), platelet-to-lymphocyte ratio (PLR), monocyte-to-lymphocyte ratio (MLR), C-reactive protein (CRP), and procalcitonin in predicting 30-day and 1-year mortality in patients undergoing femur fracture surgery.

**Materials and methods:**

This retrospective, multicenter cohort study included patients who underwent surgical treatment for femur fractures between January 2018 and December 2024. Patients were stratified into survivor and nonsurvivor groups based on 30-day and 1-year outcomes. Demographic characteristics, comorbidities, laboratory parameters, and inflammatory indices were recorded and analyzed. Linear and multivariate logistic regression analyses were performed to identify independent predictors of mortality. Receiver operating characteristic (ROC) curve analysis was used to assess the predictive performance of the inflammatory markers.

**Results:**

A total of 430 patients were included, 310 (72.1%) of whom survived while 120 (27.9%) died during the 1-year follow-up. The 30-day mortality rate was 15% (n = 65) and the 1-year mortality rate was 38% (n = 163). Nonsurvivors were significantly older (81.3 ± 9.6 vs. 75.2 ± 10.1 years, p < 0.001) and had higher rates of diabetes mellitus (45% vs. 30%, p = 0.003), hypertension (80% vs. 40%, p < 0.001), and chronic kidney disease (55% vs. 16%, p < 0.001). Laboratory analysis showed elevated white blood cell count, red cell distribution width, neutrophils, CRP, procalcitonin, NLR, PLR, and MLR, while albumin was lower in nonsurvivors (all p < 0.05). Multivariate analysis identified age, chronic kidney disease, low albumin, elevated creatinine, CRP, the MLR, and hospital stay as independent mortality predictors. The MLR showed the strongest association (OR = 4.12 for 30-day mortality; OR = 3.85 for 1-year). ROC analysis revealed CRP (AUC = 0.811 and 0.783) and the MLR (AUC = 0.765 and 0.751) as the most accurate predictors, followed by the NLR and hospital stay.

**Conclusion:**

Preoperative inflammatory markers, particularly the MLR and CRP, were strongly associated with short- and long-term mortality in femur fracture surgery. Their integration into preoperative assessment may enhance risk stratification and guide clinical decision-making.

## 1. Introduction

Femur fractures are among the most serious orthopedic injuries and are commonly seen in the elderly population. These fractures carry a substantial risk of morbidity and mortality, largely influenced by patients’ general health status, the presence of comorbidities, and their vulnerability to complications in the postoperative period [1]. In the elderly population, femur fractures often occur as a consequence of falls and are strongly associated with underlying risk factors like reduced bone density, diminished muscle strength, and balance disorders [2].

Surgical intervention is often the standard approach in the management of femur fractures, particularly for elderly patients. Given the complexity of recovery in this population, a multidisciplinary strategy is essential during the postoperative period. Equally important is a thorough preoperative evaluation of the patient’s overall health status, which plays a pivotal role in improving surgical outcomes. Recent research has increasingly highlighted the prognostic value of preoperative biochemical and inflammatory markers in estimating postoperative mortality risk [3].

Inflammation is a normal physiological reaction to trauma, infection, or surgical stress. Persistent or chronic inflammation has been shown to play a major role in the onset of postoperative complications and is closely linked to higher mortality risk in surgical patients [4]. Preoperative inflammatory markers serve as indicators of systemic inflammatory status and are commonly utilized to predict postoperative outcomes. Among these, the neutrophil-to-lymphocyte ratio (NLR), platelet-to-lymphocyte ratio (PLR), monocyte-to-lymphocyte ratio (MLR), and C-reactive protein (CRP) stand out as especially relevant parameters [5,6].

The NLR is a widely recognized marker of systemic inflammation and has emerged as a valuable prognostic indicator across a range of medical and surgical conditions. Higher NLR values are typically linked to heightened inflammatory activity and have been correlated with unfavorable clinical outcomes [7,8]. The PLR is considered an indicator of thromboinflammatory activity and has been associated with an increased risk of vascular complications [9]. The MLR reflects monocyte-driven inflammatory processes and has been utilized as a prognostic indicator in both cardiovascular conditions and various malignancies [10,11]. CRP is a well-known acute-phase protein produced by the liver and is routinely used in clinical practice to monitor the intensity and progression of inflammatory responses [12,13].

In patients undergoing femur fracture surgery, the prognostic significance of these inflammatory markers in relation to postoperative complications and mortality has not been fully established. Although some studies have indicated a potential link between elevated preoperative inflammatory levels and higher postoperative mortality, other findings have been inconclusive or contradictory [14,15].

This study aimed to investigate the influence of preoperative inflammatory markers on postoperative mortality in patients undergoing femur fracture surgery. By assessing the prognostic significance of these markers in relation to clinical outcomes, the study also intended to support the development of improved strategies for preoperative risk assessment and tailored management of high-risk individuals.

## 2. Materials and methods

### 2.1. Patients and study design

This study employed a retrospective, observational design and was conducted at a tertiary care center. It included patients who underwent surgical procedures for femur fractures in the Department of Orthopedics and Traumatology between January 2018 and December 2024. Clinical and laboratory data were obtained retrospectively through the hospital’s electronic medical records system.

The inclusion criteria comprised age of ≥18 years, being diagnosed with a femur fracture, having undergone surgical intervention, and having complete preoperative laboratory data available, including a full blood count and biochemical profile. Patients were excluded if they were under 18 years of age, received conservative treatment, or had preexisting chronic inflammatory conditions such as rheumatoid arthritis, systemic lupus erythematosus, or inflammatory bowel disease. Additional exclusion criteria included active infection, any history of malignancy, or incomplete medical records.

**Figure 1:**
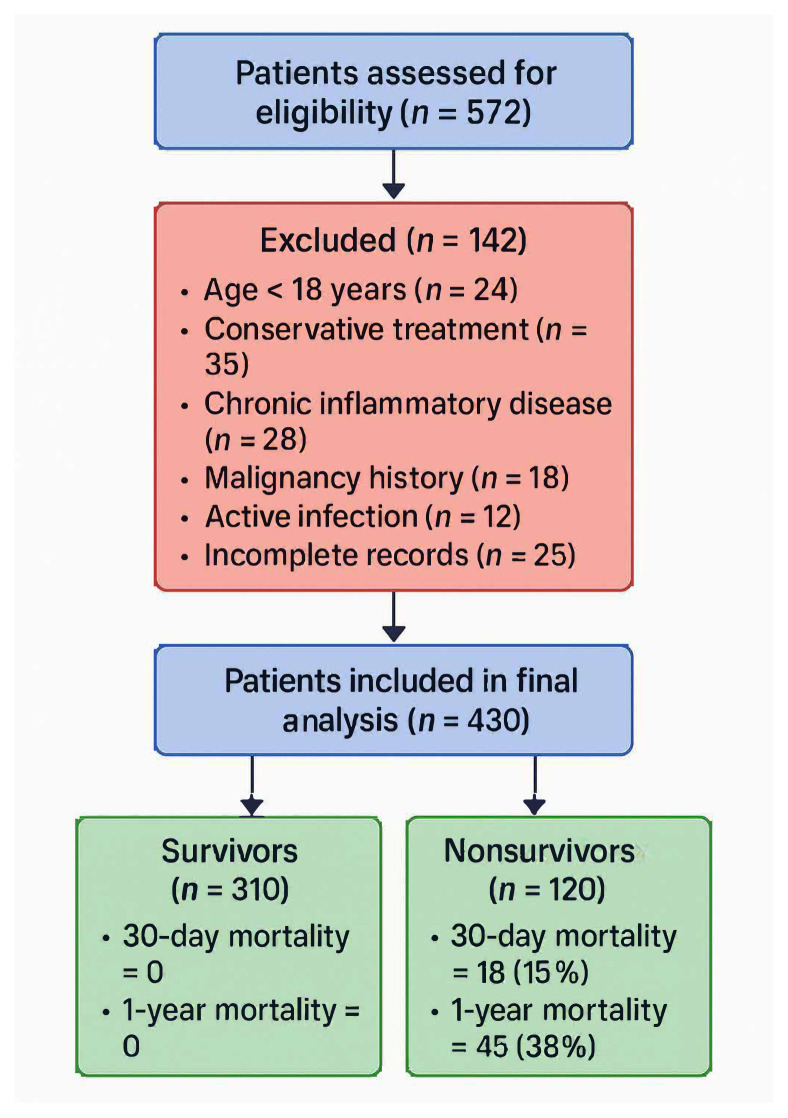
Flowchart of the study.

Patients were stratified based on their preoperative inflammatory marker levels and analyzed according to their postoperative clinical outcomes (Figure 1).

### 2.2. Data collection and laboratory analyses

The demographic data of all patients were obtained from hospital records and recorded using a standardized data collection form. The recorded variables included sex, type of fracture (proximal femur, diaphyseal, or distal femur fracture, etc.), comorbidities (diabetes mellitus, hypertension, chronic kidney disease, etc.), surgical method (internal fixation, total hip arthroplasty, etc.), intensive care unit (ICU) requirement and length of hospital stay, and 30-day and 1-year mortality rates.

Preoperative complete blood count and biochemical test results were recorded. The inflammatory markers analyzed in this study were as follows:

- Neutrophil-to-lymphocyte ratio (NLR): Calculated by dividing the neutrophil count by the lymphocyte count
- Platelet-to-lymphocyte ratio (PLR): Calculated by dividing the platelet count by the lymphocyte count
- Monocyte-to-lymphocyte ratio (MLR): Calculated by dividing the monocyte count by the lymphocyte count

Other parameters such as CRP, hemoglobin, white blood cell count (WBC), creatinine, and glucose levels were also analyzed. All laboratory tests were performed in the hospital’s biochemistry laboratory using standardized methods.

### 2.3. Statistical analysis

All statistical analyses were conducted using IBM SPSS Statistics 27.0 (IBM Corp., Armonk, NY, USA). The normality of continuous variables was assessed using the Shapiro–Wilk test. Normally distributed data were expressed as mean ± standard deviation (SD), while nonnormally distributed data were presented as median and interquartile range (IQR). Categorical variables were reported as frequencies and percentages. For comparisons of normally distributed continuous variables among groups, Welch analysis of variance (ANOVA) was employed. When significant differences were detected, the Games–Howell post hoc test was used. For nonnormally distributed continuous variables, the Kruskal–Wallis test was applied, followed by Dunn–Bonferroni post hoc tests when appropriate. Comparisons of categorical variables were performed using the chi-square test or Fisher exact test, depending on expected cell counts. To evaluate the effect of fixation type on clinical outcomes while controlling for age as a potential confounding factor, analysis of covariance (ANCOVA) was conducted. Although the Levene test indicated a violation of the assumption of homogeneity of variances for some outcome variables, results were interpreted with caution, and Bonferroni-adjusted pairwise comparisons were performed when a significant main effect was found. All outcomes were reported as age-adjusted means and standard errors (SEs). Additionally, significant differences between fixation methods were visualized using network diagrams, where nodes represented fixation types and edges indicated statistically significant relationships. Values of p < 0.05 were considered statistically significant. Effect sizes were reported using partial eta-squared (η^2^) values.

## 3. Results

Comparisons of the sociodemographic and laboratory findings of the patients are shown in Table 1. In total, 430 patients were included in the study, with 310 survivors and 120 nonsurvivors. The mean age of nonsurvivors was significantly higher than that of survivors (81.3 ± 9.6 vs. 75.2 ± 10.1 years, p < 0.001). The prevalence of comorbidities such as diabetes mellitus (45% vs. 30%, p = 0.003), hypertension (80% vs. 40%, p < 0.001), and chronic kidney disease (55% vs. 16%, p < 0.001) was notably higher among nonsurvivors. Laboratory findings revealed significantly elevated levels of WBC, red cell distribution width (RDW), neutrophils, blood urea nitrogen (BUN), creatinine, D-dimer, CRP, and procalcitonin in nonsurvivors compared to survivors (p < 0.05 for all). Conversely, survivors had higher albumin, calcium, and lymphocyte levels. Inflammatory markers including the NLR, PLR, and MLR were significantly higher in the nonsurvivor group (all p < 0.001). The mean hospital stay was longer for nonsurvivors (10.2 ± 3.4 days vs. 7.8 ± 2.6 days, p < 0.001), and ICU admission rates were also significantly higher (42% vs. 11%, p < 0.001). There was no statistically significant difference between groups in terms of fracture type (p > 0.05 for all). Early (30-day) and late (1-year) mortality rates in the overall population were 15% and 38%, respectively (Table 1).

The results of linear regression analysis for 30-day and 1-year mortality are shown in Table 2. Linear regression analysis revealed that several clinical and laboratory parameters were significantly associated with both 30-day and 1-year mortality. Age was positively correlated with mortality at both time points (p < 0.001). Among laboratory parameters, elevated levels of WBC, RDW, neutrophils, mean platelet volume (MPV), monocytes, glucose, BUN, creatinine, D-dimer, procalcitonin, CRP, and all three inflammatory ratios, including the NLR, PLR, and MLR, were significantly associated with increased risk of mortality in both the early and late postoperative periods (p < 0.05 for all). Conversely, lower levels of albumin, calcium, and lymphocyte count were negatively associated with mortality, indicating a potential protective role. In addition, longer surgery duration and extended length of hospital stay were significantly related to increased mortality risk at both 30 days and 1 year (p < 0.05 for all) (Table 2).

Multiple logistic regression analysis results for 30-day and 1-year mortality are shown in Table 3. Multivariate logistic regression analysis identified several independent predictors of both 30-day and 1-year postoperative mortality. Increasing age was significantly associated with higher mortality risk [odds ratio (OR) = 1.08 and 1.07, respectively; p < 0.001)]. The presence of comorbidities such as diabetes mellitus, hypertension, and chronic kidney disease was also independently related to increased mortality, with chronic kidney disease showing the highest risk (OR = 2.45 for 30-day and 2.28 for 1-year mortality; p < 0.001 for both). Among laboratory parameters, elevated serum creatinine, CRP, and inflammatory markers including the NLR, PLR, and MLR were significantly associated with increased mortality. Notably, the MLR was the strongest inflammatory predictor, with an OR value of 4.12 for 30-day and 3.85 for 1-year mortality (p < 0.001 for both). Low albumin levels were found to be protective, significantly reducing mortality risk (OR = 0.62 and 0.65; p < 0.001). In addition, longer surgery duration and extended length of hospital stay were independently associated with higher postoperative mortality at both time points (p < 0.05) (Table 3).

**Figure 2:**
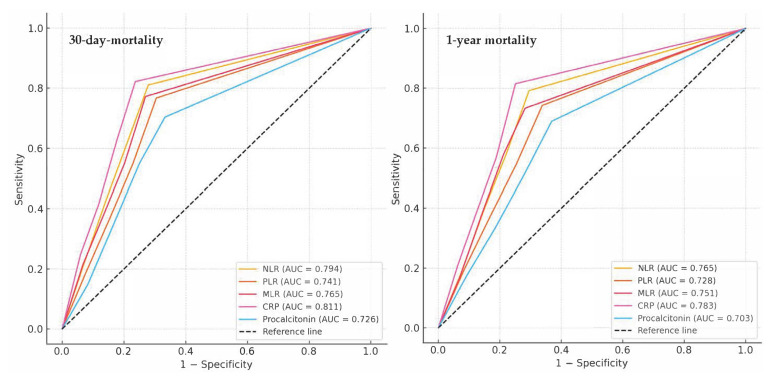
ROC analysis of inflammatory markers for 30-day and 1-year mortality.

Receiver operating characteristic (ROC) curve analysis results for inflammatory markers and 30-day and 1-year mortality are shown in Table 4 and Figure 2. All preoperative inflammatory markers demonstrated statistically significant predictive power for both 30-day and 1-year mortality (all p < 0.001). Among them, CRP showed the highest discriminative ability for 30-day mortality [area under the ROC curve (AUC): 0.811; sensitivity: 84.2%; specificity: 76.3), followed by the MLR (AUC: 0.765), NLR (AUC: 0.794), and PLR (AUC: 0.741). Although procalcitonin was also statistically significant, it had a relatively lower AUC (0.726). A similar trend was observed for 1-year mortality, with CRP (AUC: 0.783) and the MLR (AUC: 0.751) exhibiting the strongest predictive performance. Other markers, including the NLR (AUC: 0.765) and PLR (AUC: 0.728), also showed meaningful predictive accuracy (Table 4; Figure 2).

## 4. Discussion

In this retrospective cohort study, we evaluated the prognostic value of preoperative inflammatory markers and clinical parameters in predicting 30-day and 1-year mortality in patients undergoing surgery for femur fractures. Our findings revealed that age, presence of comorbidities (particularly chronic kidney disease), elevated inflammatory indices (NLR, PLR, MLR, CRP), and prolonged hospital stay were all independently associated with increased postoperative mortality. Among the inflammatory markers, the MLR emerged as the strongest predictor, followed by CRP and the NLR.

Our results are consistent with prior studies that have highlighted the prognostic utility of inflammatory biomarkers in surgical and trauma patients. Chen et al. demonstrated that elevated NLR levels are associated with poor clinical outcomes in elderly patients with hip fractures and they identified NLR as an independent predictor of 1-year mortality [16]. Wang et al. reported that higher preoperative NLR and PLR levels significantly increased the risk of postoperative complications and mortality in geriatric patients undergoing orthopedic surgery [17]. While most previous studies have focused primarily on the NLR or CRP alone, our study provides a comparative analysis of multiple inflammatory indices, including the MLR and PLR, in a single model, enhancing its clinical relevance.

In the present study, CRP was also significantly associated with both short- and long-term mortality. This aligns with findings by Niessen et al., who observed that elevated CRP levels in the perioperative period were strongly predictive of mortality and prolonged recovery following hip fracture surgery [18]. Moreover, the high sensitivity and specificity of CRP in our ROC analysis (AUC: 0.811 for 30-day and 0.783 for 1-year mortality) underscore its value as a reliable inflammatory marker.

Of particular interest, the MLR had the highest OR value among all biomarkers in our logistic regression model (OR = 4.12 for 30-day mortality and OR = 3.85 for 1-year mortality), suggesting a strong association between monocyte-mediated inflammation and adverse outcomes. Bingol et al. emphasized the role of the MLR as a potent marker in predicting mortality and systemic stress response after major orthopedic trauma [19].

In terms of comorbid conditions, our results reaffirm the significant impact of chronic kidney disease, diabetes mellitus, and hypertension on mortality risk, consistent with prior studies such as that by Barbosa et al., who reported that impaired renal function is a major determinant of postoperative mortality in elderly patients undergoing femur fracture surgery [20].

Another key finding was the association between length of hospital stay and increased mortality, which may reflect greater clinical instability or postoperative complications. This observation is also supported by Merrell et al., who noted that prolonged hospitalization is linked to frailty and poor outcomes in elderly fracture patients [21].

Compared to previously published studies, our research provides a more comprehensive analysis by simultaneously evaluating multiple inflammatory markers, including the NLR, PLR, MLR, CRP, and procalcitonin, alongside clinical and biochemical parameters such as albumin, creatinine, and hospital stay duration. While most earlier investigations have focused on the predictive value of a single biomarker, particularly the NLR or CRP, for short-term mortality [22,23], our study incorporates both 30-day and 1-year outcomes in a multivariate logistic regression model. Additionally, we identified the MLR as the strongest independent inflammatory predictor of mortality, a parameter that has received limited attention in the context of femur fracture surgery [24]. Furthermore, the inclusion of length of hospital stay as a significant prognostic factor provides new insight into perioperative management, a factor often overlooked in prior analyses [25–27]. By integrating a broader set of markers and extending the mortality assessment beyond the acute period, our study offers a more robust risk stratification framework for clinical practice.

This study has some limitations. First, its retrospective design may introduce selection and information biases, as data were collected from existing medical records. Second, while we included a wide range of inflammatory and biochemical markers, potential confounding variables such as nutritional status, medication use, and postoperative complications could not be fully accounted for due to incomplete data. Additionally, the timing of preoperative blood sampling may have varied slightly among patients, potentially influencing the levels of inflammatory markers. Furthermore, although we examined both 30-day and 1-year mortality outcomes, we did not evaluate functional outcomes or quality of life measures after surgery, which could have provided a more holistic view of patient prognosis. Finally, cause-specific mortality was not analyzed, which limits our ability to differentiate deaths due to surgical complications from those related to comorbid conditions. Despite these limitations, the study provides valuable insights into the prognostic utility of preoperative inflammatory markers in femur fracture surgery and highlights potential targets for risk stratification and early intervention.

## 5. Conclusion

This study has demonstrated that preoperative inflammatory markers including the NLR, PLR, MLR, CRP, and procalcitonin were significantly associated with both 30-day and 1-year mortality in patients who underwent surgery for femur fractures. Among these markers, the MLR and CRP emerged as the most powerful predictors, indicating a strong association between systemic inflammation and adverse postoperative outcomes. Additionally, older age, the presence of comorbidities such as chronic kidney disease, lower albumin levels, elevated creatinine, and longer hospital stays were identified as independent risk factors for mortality. These findings suggest that routine evaluation of inflammatory and biochemical parameters during the preoperative period could improve risk stratification and perioperative management. Incorporating such markers into clinical protocols might enable clinicians to better identify high-risk patients who require closer monitoring and more individualized care. Further prospective and multicenter studies are warranted to confirm these results and to determine standardized cut-off values for broader clinical application.

## Figures and Tables

**Table 1:** Comparison of sociodemographic and laboratory findings of patients (N = 430).

Variable	Survivors (n=310)	Nonsurvivors (n=120)	p-value
	Mean ± SD or n (%)		
Age (years)	75.2 ± 10.1	81.3 ± 9.6	<0.001
Sex (male/female)	174 / 136	66 / 54	0.178
Diabetes mellitus	93 (30%)	54 (45%)	0.003
Hypertension	124 (40%)	96 (80%)	<0.001
Chronic kidney disease	50 (16%)	66 (55%)	<0.001
WBC (×10^9^/μL)	8.2 ± 2.1	9.4 ± 2.6	0.002
RDW (%)	13.5 ± 1.4	14.9 ± 2.0	0.012
Platelet (×10^9^/μL)	210 ± 55	195 ± 60	0.087
Neutrophil (×10^9^/μL)	5.1 ± 1.3	6.8 ± 1.7	<0.001
MPV (fL)	9.1 ± 1.2	10.5 ± 1.3	0.009
Monocyte (×10^9^/μL)	0.62 ± 0.19	0.78 ± 0.25	0.031
Lymphocyte (×10^9^/μL)	2.1 ± 0.8	1.4 ± 0.6	0.045
Ca++ (mg/dL)	9.2 ± 0.4	8.8 ± 0.6	0.005
Glucose (mg/dL)	110 ± 25	135 ± 30	<0.001
Albumin (g/dL)	3.6 ± 0.6	2.8 ± 0.7	<0.001
BUN (mmol/L)	14.2 ± 6.3	19.8 ± 7.2	<0.001
Creatinine (mg/dL)	1.0 ± 0.3	1.4 ± 0.4	<0.001
D-dimer (mg/L)	1.2 ± 0.8	2.9 ± 1.0	<0.001
C-reactive protein (CRP) (mg/L)	13.5 ± 5.9	22.7 ± 6.7	<0.001
Neutrophil-to-lymphocyte ratio (NLR)	2.8 ± 1.1	4.9 ± 1.6	<0.001
Platelet-to-lymphocyte ratio (PLR)	120 ± 35	178 ± 42	<0.001
Monocyte-to-lymphocyte ratio (MLR)	0.31 ± 0.09	0.54 ± 0.13	<0.001
Procalcitonin (ng/L)	0.07 ± 0.02	0.13 ± 0.03	0.014
Fracture type			
Proximal femur fracture	217 (70%)	84 (70%)	0.611
Diaphyseal fracture	62 (20%)	18 (15%)	0.223
Distal femur fracture	31 (10%)	18 (15%)	0.998
Surgery type (internal/arthroplasty)	186/124	78/42	0.044
Surgery duration (min)	85 ± 20	95 ± 25	0.021
ICU admission	34 (11%)	50 (42%)	<0.001
Length of hospital stay (days)	7.8 ± 2.6	10.2 ± 3.4	<0.001
Mortality			
30-day	-	18 (15%)	<0.001
1-year	-	45 (38%)	<0.001

**Table 2:** Linear regression analysis for 30-day and 1-year mortality.

Parameter	β Coefficient (30-day)	p-value	β Coefficient (1-year)	p-value
Age	0.215	<0.001	0.198	<0.001
WBC (×10^9^/μL)	0.210	0.002	0.194	0.003
RDW (%)	0.158	0.010	0.143	0.012
Platelet (×10^9^/μL)	−0.075	0.083	−0.068	0.091
Neutrophil (×10^9^/μL)	0.226	<0.001	0.209	<0.001
MPV (fL)	0.135	0.014	0.126	0.017
Monocyte (×10^9^/μL)	0.201	<0.001	0.185	<0.001
Lymphocyte (×10^9^/μL)	−0.187	0.005	−0.172	0.007
Ca++ (mg/dL)	−0.120	0.037	−0.109	0.041
Glucose (mg/dL)	0.239	<0.001	0.221	<0.001
Albumin (g/dL)	−0.330	<0.001	−0.312	<0.001
BUN (mmol/L)	0.285	<0.001	0.268	<0.001
Creatinine (mg/dL)	0.198	<0.001	0.182	<0.001
D-dimer (mg/L)	0.251	<0.001	0.236	<0.001
Procalcitonin (ng/L)	0.179	0.014	0.163	0.018
C-reactive protein (CRP) (mg/L)	0.310	<0.001	0.296	<0.001
Neutrophil-to-lymphocyte ratio (NLR)	0.295	<0.001	0.278	<0.001
Platelet-to-lymphocyte ratio (PLR)	0.271	<0.001	0.254	<0.001
Monocyte-to-lymphocyte ratio (MLR)	0.288	<0.001	0.267	<0.001
Surgery duration (min)	0.111	0.019	0.105	0.022
Length of hospital stay (days)	0.174	<0.001	0.162	<0.001

**Table 3:** Multiple logistic regression analysis for 30-day and 1-year mortality.

Parameter	30-day mortality	p-value	1-year mortality	p-value
	OR (95% CI)		OR (95% CI)	
Age	1.08 (1.04–1.13)	<0.001	1.07 (1.03–1.11)	<0.001
Diabetes mellitus	1.72 (1.15–2.58)	0.008	1.64 (1.14–2.35)	0.012
Hypertension	1.88 (1.30–2.74)	<0.001	1.72 (1.25–2.38)	<0.001
Chronic kidney disease	2.45 (1.68–3.58)	<0.001	2.28 (1.62–3.23)	<0.001
Albumin (g/dL)	0.62 (0.48–0.79)	<0.001	0.65 (0.52–0.82)	<0.001
Creatinine (mg/dL)	1.90 (1.32–2.75)	<0.001	1.76 (1.28–2.41)	<0.001
CRP (mg/L)	1.12 (1.07–1.18)	<0.001	1.09 (1.05–1.14)	<0.001
Neutrophil-to-lymphocyte ratio (NLR)	1.65 (1.30–2.09)	<0.001	1.58 (1.28–1.95)	<0.001
Platelet-to-lymphocyte ratio (PLR)	1.01 (1.00–1.02)	0.032	1.01 (1.00–1.02)	0.038
Monocyte-to-lymphocyte ratio (MLR)	4.12 (1.85–9.20)	<0.001	3.85 (1.79–8.30)	<0.001
Surgery duration (min)	1.03 (1.00–1.06)	0.041	1.02 (1.00–1.05)	0.048
Length of hospital stay (days)	1.21 (1.12–1.31)	<0.001	1.18 (1.10–1.26)	<0.001

**Table 4:** ROC analysis of inflammatory markers for 30-day and 1-year mortality.

Parameters	Cut-off	Sensitivity	Specificity	AUC (95% CI)	p-value
**30-day mortalit**y					
NLR	3.6	81.3	72.1	0.794 (0.740–0.849)	<0.001
PLR	150	76.0	69.5	0.741 (0.682–0.800)	<0.001
MLR	0.40	78.5	73.0	0.765 (0.710–0.820)	<0.001
CRP (mg/L)	18.0	84.2	76.3	0.811 (0.760–0.862)	<0.001
Procalcitonin (ng/L)	0.09	70.4	66.7	0.726 (0.670–0.782)	<0.001
**1-year mortalit**y					
NLR	3.4	78.2	70.5	0.765 (0.710–0.820)	<0.001
PLR	145	73.5	66.2	0.728 (0.668–0.788)	<0.001
MLR	0.38	75.0	71.8	0.751 (0.696–0.807)	<0.001
CRP (mg/L)	17.0	80.6	74.9	0.783 (0.729–0.838)	<0.001
Procalcitonin (ng/L)	0.08	67.5	63.1	0.703 (0.646–0.760)	<0.001
